# Correction: LCK’ed in: Inborn errors of immunity in LCK reveal how TCR signaling is calibrated

**DOI:** 10.1084/jem.2024104606082026c

**Published:** 2026-06-24

**Authors:** Ahmet Eken, Sara A. Johnson, Serife Erdem, Elena W.Y. Hsieh

Vol. 223, No. 6 | https://doi.org/10.1084/jem.20241046 | May 5, 2026

The authors regret that, in their originally published review, the MHC II molecule in Fig. 2 B was inaccurately portrayed as MHC I. This error was made during figure preparation. The original and corrected figures are shown here. This correction does not change the original conclusions of the review, and the figure legend remains unchanged. The HTML and PDF versions of this review have been corrected. The error remains only in print and in PDFs downloaded before June 16, 2026.

**Figure:**
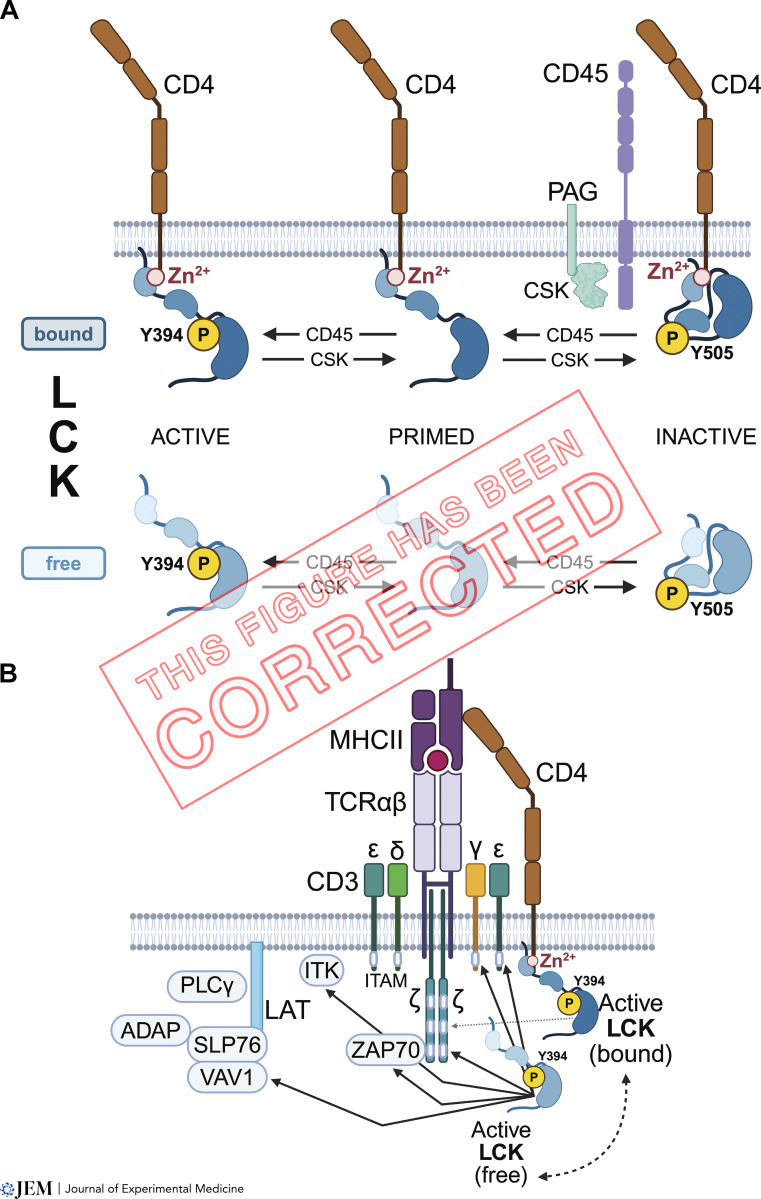


**Figure 2.**
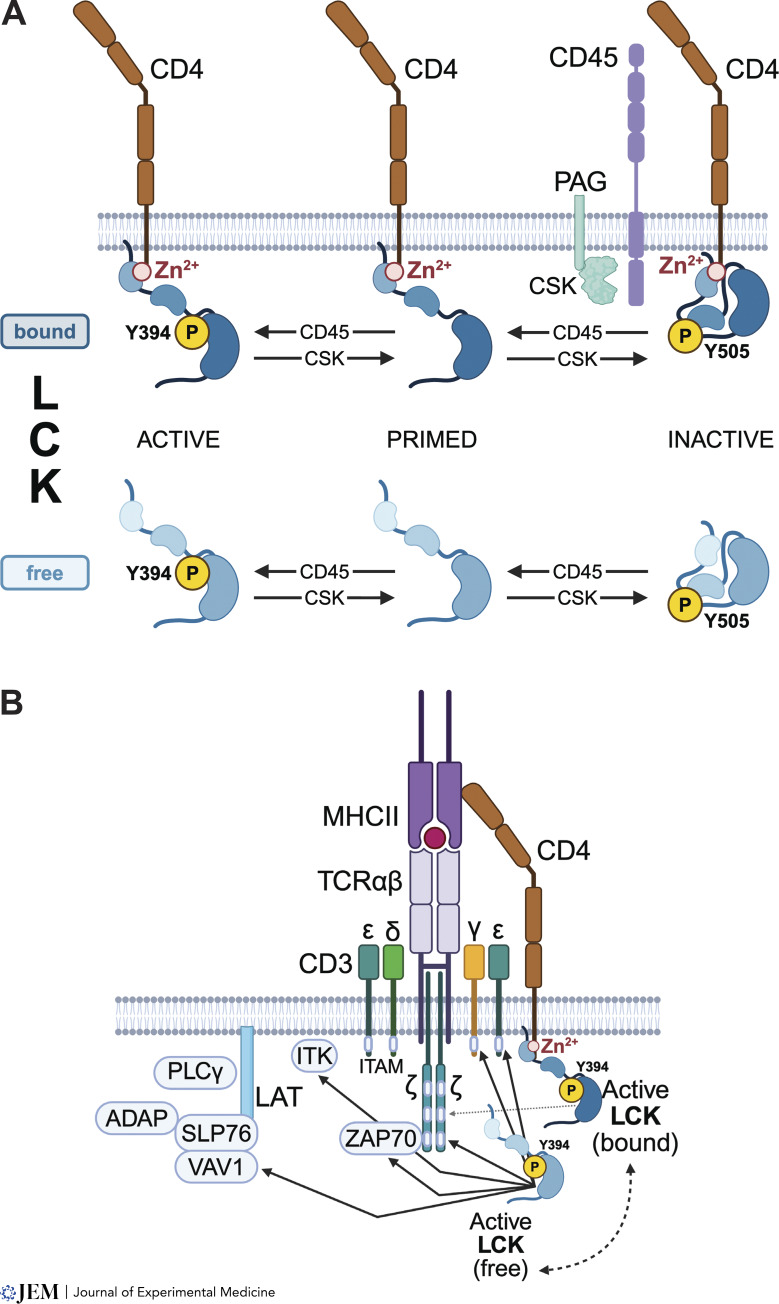
**Regulation and signaling functions of LCK in proximal TCR signaling. (A)** LCK exists in co-receptor–bound and free pools, each cycling among active, primed, and inactive conformations. CD45 dephosphorylates the inhibitory Y505, promoting activation, whereas CSK (recruited via PAG) phosphorylates Y505 to maintain the inactive state. Autophosphorylation of Y394 stabilizes the active conformation. Zn^2+^ coordinates LCK binding to CD4/CD8 co-receptors. **(B)** Upon TCR engagement, the free, active pool of LCK initiates TCR signaling via phosphorylation of ITAMs within CD3 chains, while co-receptor–bound LCK modulates sensitivity, efficiency, and lineage calibration. ITAM phosphorylation enables ZAP-70 recruitment and activation of downstream effectors, including LAT, SLP-76, and ITK.

